# The ClpP Protease Is Required for the Stress Tolerance and Biofilm Formation in *Actinobacillus pleuropneumoniae*

**DOI:** 10.1371/journal.pone.0053600

**Published:** 2013-01-11

**Authors:** Fang Xie, Yanhe Zhang, Gang Li, Long Zhou, Siguo Liu, Chunlai Wang

**Affiliations:** State Key Laboratory of Veterinary Biotechnology, Division of Bacterial Diseases, Harbin Veterinary Research Institute, Chinese Academy of Agricultural Sciences, Harbin, People's Republic of China; Miami University, United States of America

## Abstract

In the respiratory tract and lung tissue, a balanced physiological response is essential for *Actinobacillus pleuropneumoniae* to survive various types of challenges. ClpP, the catalytic core of the Clp proteolytic complex, is involved in various stresses response and regulation of biofilm formation in many pathogenic bacteria. To investigate the role of ClpP in the virulence of *A. pleuropneumoniae*, the *clpP* gene was deleted by homologous recombination, resulting in the mutant strain S8*ΔclpP*. The reduced growth of S8*ΔclpP* mutant at high temperatures and under several other stress conditions suggests that the ClpP protein is required for the stress tolerance of *A. pleuropneumoniae*. Interestingly, we observed that the S8*ΔclpP* mutant exhibited an increased ability to take up iron in vitro compared to the wild-type strain. We also found that the cells without ClpP displayed rough and irregular surfaces and increased cell volume relative to the wild-type strain using scanning electron microscopy (SEM). Confocal laser scanning microscopy (CLSM) revealed that the S8*ΔclpP* mutant showed decreased biofilm formation compared to the wild-type strain. We examined the transcriptional profiles of the wild type S8 and the S8*ΔclpP* mutant strains of *A. pleuropneumoniae* using RNA sequencing. Our analysis revealed that the expression of 16 genes was changed by the deletion of the *clpP* gene. The data presented in this study illustrate the important role of ClpP protease in the stress response, iron acquisition, cell morphology and biofilm formation related to *A. pleuropneumoniae* and further suggest a putative role of ClpP protease in virulence regulation.

## Introduction

*Actinobacillus pleuropneumoniae* is the causative agent of porcine pleuropneumonia, a highly contagious and often fatal respiratory disease that affects pigs worldwide [1]. This organism can cause sudden death and can colonize the respiratory tracts, tonsils and lungs of pigs, causing chronic and persistent infections, lung lesions, and reduced growth [2]. The ability of *A. pleuropneumoniae* to persist in host tissues is a major obstacle to the eradication of the organism [1], [3], [4], which is the primary source for new cases. Moreover, the disease causes serious economic losses for the swine industry [5].

Transitioning between respiratory tract and lung tissue subjects *A. pleuropneumoniae* to environmental stresses. *A. pleuropneumoniae* is well equipped to respond to these stressors through the production of a series of stress-related proteins [6]. Among these proteins, the ClpP protease, which is the member of the Clp (caseinolytic protease, Hsp100) family, has been studied in several pathogenic bacteria and has proved to be an important virulence factor [7]–[15].

The ClpP protease was first discovered and is best characterized in *Escherichia coli* [16], [17]. ClpP protease is important for normal growth and is involved in the stress response and the degradation of misfolded proteins in most bacteria, including *E. coli* and *Salmonella enterica* [18], [19]. Clp proteolytic enzymes are also required for full virulence in several pathogenic organisms, including *Listeria monocytogenes*, *Yersinia pestis*, *Mycobacterium tuberculosis* and *Helicobacter pylori* [7]–[10]. Interestingly, the ClpP proteases may affect biofilm formation in some bacteria. Decreased biofilm formation was observed in *clpP* mutants of *Pseudomonas fluorescens*, *Streptococcus mutans* and *Staphylococcus epidermidis* [11]–[13], while increased biofilm formation was observed in *clpP* mutants of *Staphylococcus aureus* and P*seudomonas aeruginosa* [14], [15]. There is, however, no evidence that ClpP protease plays a role in the stress response or biofilm formation related to *A. pleuropneumoniae*.

In the present study, we inactivated the *clpP* gene in *A. pleuropneumoniae* strain S8 by homologous recombination using a sucrose counter-selectable marker system. We found that the ClpP protease mediates tolerance to multiple stressors, including heat, oxidative stress and osmotic stress. Interestingly, we found that the deletion of the *clpP* gene improved the iron utilization of *A. pleuropneumoniae*. We also showed that ClpP affects the cell morphology of and biofilms formed by *A. pleuropneumoniae* and that ClpP might play an important role in the regulation of virulence.

## Materials and Methods

### Bacterial strains, plasmids and growth conditions

The bacterial strains, plasmids and primers used in this work are listed in Table 1. *E. coli* β2155 (*ΔdapA*) was cultured in LB medium supplemented with diaminopimelic acid (1 mM; Sigma-Aldrich, St. Louis, MO, USA). *A. pleuropneumoniae* serotype 7 strain S8 was isolated from the lung of a diseased pig in northern China. *A. pleuropneumoniae* strains were cultured in brain heart infusion (BHI, Oxoid Ltd, Basingstoke, Hampshire, UK) supplemented with nicotinamide dinucleotide (NAD, 10 µg/mL; Sigma-Aldrich, St. Louis, MO, USA). *A. pleuropneumoniae* transconjugants (single crossovers) and transformants were grown in a BHI supplemented with NAD (10 µg/mL) and chloramphenicol (5 µg/mL). *A.pleuropneumoniae* S8HB were grown in a BHI supplemented with NAD (10 µg/mL) and kanamycin (30 µg/mL).

**Table 1:** Characteristics of bacterial strains, plasmids, and primers used in this study.

Strains, plasmids, and primers	Characteristics or sequence	Source or reference
**Strains**		
*E. coli* β2155	*thrB1004 pro thi strA hsdS lac*ZΔM15 (F′ *lacZ*ΔM15 *lacl* ^q^ *traD36 proA* ^+^ *proB* ^+^)Δ*dap* :: *erm* (Erm^r^))*recA* :: *RPA-2-tet* (Tc^r^)::Mu-km (Km^r^) λ*pir*	[21]
*A.pleuropneumoniae* S8	*A. pleuropneumoniae* serotype 7 clinical isolate from the lung of a diseased pig in northern China	This work
*A.pleuropneumoniae* S8*ΔclpP*	Unmarked ClpP protease-negative knockout mutant of *A. pleuropneumoniae* S8	This work
*A.pleuropneumoniae* S8HB	The complemented strain of *A. pleuropneumoniae* S8*ΔclpP* containing the *clpP* gene	This work
**Plasmids**		
pMD18-T simple	A cloning vector	Takara
pMD*ΔclpP*	Cloning vector with a 491 bp deletion in the *clpP* gene which have a 1.2-kb upstream fragment and 1.2-kb downstream fragment	This work
pEMOC2	Conjugative vector based on pBluescript SK with mob RP4, polycloning site, *Cm^r^*, and transcriptional fusion of the omlA promoter with the *sacB* gene	Accession no. AJ868288, [22]
pEM*ΔclpP*	Conjugative vector pEMOC2 with a 491 bp deletion in the *clpP* gene which have a 1.2-kb upstream fragment and 1.2-kb downstream fragment	This work
pLS88	Broad-host-range shuttle vector from *Haemophilus ducreyi*; Str^r^ Sm^r^ Km^r^	[24]
pLSclpP	pLS88 with a PCR-derived insert containing the *clpP* gene	This work
**Primers**		
clpPSF	5′ GCGTCGACGGGGCGTTACTGGATGC 3′, upstream primer with internal SalI site (underlined) comprising positions 1157 to 1141 upstream of the *clpP* gene start codon	This work
clpPSR	5′ CCATCGCTTC **CGCCTTTGGA**GGTTTGC 3′, downstream primer with reverse complement sequence(underlined) of sequence in bold from primer clpPXF, comprising positions 24 to 40 downstream of the *clpP* gene start codon	This work
clpPXF	5′ TCCAAAGGCG **GAAGCGATGG**AATACGGTC 3′, upstream primer with reverse complement sequence(underlined) of sequence in bold from primer clpPSR, comprising positions 54 to 36 upstream of the *clpP* gene stop codon	This work
clpPXR	5′ TTGCGGCCGCTTCTCTGCTTTAAGTGTCGGC 3′, downstream primer with internal NotI site (underlined) comprising positions 1172 to 1192 downstream of the *clpP* gene stop codon	This work
clpPJDF	5′ CGTGGTGTCGCTTGAAACTC 3′, upstream primer comprising positions 300 to 281 upstream of the *clpP* gene start codon	This work
clpPJDR	5′ AATTAGACCGTATTCCATCGC 3′, downstream primer comprising positions 51 to 31 upstream of the *clpP* gene stop codon	This work
clpPHBF	5′ CGGAATTCATGGCATTAGTACCAATAGTG 3′, upstream primer with internal *Eco*RI site (underlined) comprising positions 1 to 21 downstream of the *clpP* gene start codon	This work
clpPHBR	5′ CGAGCTCTTATTTTATATCTCTGTGTGTTA 3′, downstream primer with internal *Sac*I site (underlined) comprising positions 23 to 1 upstream of the *clpP* gene stop codon	This work

### Chromosomal inactivation of the clpP gene

Primers clpPSF/clpPSR and clpPXF/clpPXR (Table 1) were designed to generate a 491 bp internal deletion in the *clpP* gene by single-overlap extension PCR (SOE PCR) [20]. The resultant 2449 bp PCR product, containing an internal in-frame deletion of 491 bp in the *clpP* gene (from nt 44 to 534), was ligated into the conjugative plasmid pEMOC2 to yield plasmid pEM*ΔclpP*. Using *E. coli* strain β2155 [21], plasmid pEM*ΔclpP* was used to introduce the *clpP* deletion into *A. pleuropneumoniae* strain S8 via the single-step transconjugation system, as described previously [22], [23], resulting in *A. pleuropneumoniae* S8*ΔclpP*. PCR using primers clpPJDF/clpPJDR was used to distinguish between wild-type strains and mutants, and the PCR products were sequenced.

### Complementation of *A. pleuropneumoniae* S8*ΔclpP*

The pLSclpP plasmid was constructed by cloning the 591-bp PCR product generated by the clpPHBF/clpPHBR primers (Table 1), which contained the entire *clpP* open reading frame (ORF), into the pLS88 plasmid [24]. The plasmid was then electroporated into *A. pleuropneumoniae* strain S8*ΔclpP*. The complemented *A. pleuropneumoniae* mutants were selected on BHI containing both NAD and kanamycin, and confirmed with PCR using the clpPHBF/clpPHBR primers.

### Growth experiments

*A. pleuropneumoniae* the wild-type S8 strain, the S8*ΔclpP* mutant and the complemented S8HB strain were first grown in 5 ml of BHI for about 20 h and then diluted to similar optical densities at an OD_600_ value of approximately 0.2. These new cultures were then incubated at 25°C, 37°C and 42°C. OD_600_ was determined using an Eppendorf Biophotometer (Eppendorf, Hamburg, Germany) at various time points. The experiments were carried out in triplicate.

### Stress resistance assays

Stress resistance assays were conducted using *A. pleuropneumoniae* wild-type S8 strain, the S8*ΔclpP* mutant and the complemented S8HB strain. These strains were grown in BHI at 37°C. At an OD_600_ value of approximately 0.6, cells from 1 ml of broth cultures were centrifuged at 5,000 g for 5 min. For the heat-shock assay, cells were resuspended in BHI and placed in a 52°C water bath for 20 min. For the oxidative tolerance assay, the cells were resuspended in 1 ml of BHI supplemented with 1 mM hydrogen peroxide for 30 min. For the osmotic tolerance assay, the cells were resuspended in 1 ml of BHI supplemented with 0.3 M potassium chloride for 1 h. The control samples of each strain were resuspended in 1 ml of BHI without any treatment. Then, the cultures from each stress resistance assay were serially diluted in BHI, and spread on BHI plates for CFU counting. Stress resistance was calculated as [(stressed sample CFU ml^−1^)/(control sample CFU ml^−1^)]×100. The experiments were carried out in triplicate.

### Iron utilization assays

The iron utilization assay has been described previously [25], [26]. Briefly, *A. pleuropneumoniae* strains S8, S8*ΔclpP* and S8HB were grown in 5 ml of BHI for about 20 h and then diluted into fresh BHI medium containing 30 µM of the iron chelator ethylenediamine dihydroxyphenylacetic acid (EDDHA) to similar optical densities at an OD_600_ value of approximately 0.2. In the iron supplementation culture, 10 µM FeSO_4_ was added to the iron chelated culture after the addition of EDDHA. OD_600_ was determined using an Eppendorf Biophotometer (Eppendorf, Hamburg, Germany) at various time points. The experiments were carried out in triplicate.

### Scanning electron microscopy

For scanning electron microscopy, *A. pleuropneumoniae* strains S8, S8*ΔclpP* and S8HB were grown to similar optical densities at the OD_600_ value of approximately 0.5, 2.0 and 2.7, respectively. Then washed 3 times with phosphate-buffered saline (PBS; in mM: 137 NaCl, 2.7 KCl, 1.8 KH_2_PO_4_ and 10 Na_2_HPO_4_, pH 7.4). Cultures were then fixed with 2.5% glutaraldehyde and 1% OsO_4_, dehydrated in a graded ethanol series and embedded in isoamyl acetate. The cells were dried using a critical point drying method, mounted on aluminum stubs and shadowed with gold. A scanning electron microscope (S-3400N, Hitachi, Japan) at 5 kV was used to visualize cells.

### Polystyrene microtiter plate biofilm assay

The microtiter plate biofilm assay is a static assay that is particularly useful for examining biofilm formation [27]. The wells of a sterile, 96-well microtiter plate (Costar^@^3599, Corning, NY, USA) were filled in triplicate with a dilution (1/100) of an overnight bacterial culture. Following an incubation period of 8–48 h at 37°C, the wells were washed with water and stained with crystal violet, as previously described [11]. The bound dye was solubilized in 100 µL of 70% ethanol, and the optical density of the ethanol-dye solution was measured using a microplate reader (Bio-Tek Instruments, Winooski, VT, USA).

### Confocal laser scanning microscopy

The same biofilm assay protocol was used as mentioned above. After 16 h of incubation, the wells were washed with water to remove non-adherent bacteria. Cells were then stained with LIVE/DEAD^@^ BacLight^Tm^ Bacterial Viability Kit solution (Molecular Probes, Eugene, Oregon, USA), incubated for 20 min at room temperature in the dark, and washed with water. The plate was examined with a confocal microscope (TCS SP5, Leica Microsystems, Hamburg, Germany). SYTO 9 nucleic acid stain was excited at 488 nm and detected using 520 nm filters. Propidium iodide was excited at 488 nm and detected using 572 nm filters.

### RNA isolation, cDNA library construction and sequencing

*A. pleuropneumoniae* strains S8 and S8*ΔclpP* were grown to early log phase (OD_600_ nm = 0.5) in BHI. The cells were collected at 4°C, and the RiboPure-Bacteria kit (Ambion) was used according to the manufacturer's instructions to isolate RNA. Both of the samples were quantified and examined for protein and reagent contamination with a Nanodrop ND-1000 spectrophotometer (NanoDrop, Wilmington, DE, USA). The RNA samples exhibiting a 23S/16S rRNA band intensity of 2∶1, a spectroscopic A260/A280 nm ratio of 1.8–2.0, and an A260/A230 nm ratio greater than 1.5 were selected for analysis. A total of 20 µg of RNA was equally pooled from the S8 and S8*ΔclpP* strains for cDNA library preparation.

Illumina sequencing was performed at the Beijing Genomics Institute (BGI)-Shenzhen in Shenzhen, China (http://www.genomics.cn/index) according to the manufacturer's instructions (Illumina, San Diego, CA). The cDNA libraries were prepared according to Illumina's protocols and sequenced using the Illumina HiSeq 2000.

### Differential expression analysis

The raw sequence reads were filtered by the Illumina pipeline. All of the low-quality reads, reads with adaptor contamination, and reads with a copy number of one were excluded from the analysis, while the clean reads were mapped to the *A. pleuropneumoniae* serotype 7 strain S8 reference sequences (Genbank accession No. ALYN00000000.1).

To identify the genes affected by the deletion of the *clpP* gene, the libraries were initially compared. To complete this procedure, the number of reads for each coding region was determined, the number of total reads was normalized between the libraries and the ratio of S8 reads to S8*ΔclpP* reads was calculated. The differentially expressed genes were detected as previously described [28], with the false discovery rate (FDR) being set below 0.01 [29]. An FDR≤0.001 and a log2Ratio absolute value ≥1 was set as the threshold for significant differences in gene expression.

The Blast2GO program [30] was used to obtain GO annotations for molecular functions, biological processes and cellular component ontologies (http://www.geneontology.org). The Kyoto Encyclopedia of Genes and Genomes pathway database [31] (http://www.genome.jp/kegg) was used to make pathway assignments. The BlastN program (http://blast.ncbi.nlm.nih.gov/) was also used to compare these sequences to the *A. pleuropneumoniae* serotype 7 strain AP76 reference sequences (Genbank accession No. CP001091.1) and obtain a historical annotation.

### Statistical analysis

Basic statistical analyses were conducted using the SPSS software (SPSS, Inc., Chicago, IL, USA). The Student's t test was used to determine the significance of the differences in the means between multiple experimental groups. The data were expressed as the mean +/− standard deviation, and values of *P*<0.05 were considered to be significant.

## Results

### Construction of S8*ΔclpP* mutant strain

In order to determine whether the function of ClpP protease was crucial for the stress tolerance and biofilm formation related to *A. pleuropneumoniae*, we constructed an isogenic *clpP* deletion mutant of *A. pleuropneumoniae* S8 with plasmid pEMOC2. The *clpP* deletion mutant was constructed by the allelic exchange of the wild-type *clpP* gene with an in-frame deletion lacking 491 bp at position 44–534 of the *clpP* ORF (Figure S1 and Figure S2). The resulting *A. pleuropneumoniae clpP* mutant was designated as S8*ΔclpP*.

### Growth experiments

**Figure 1:**
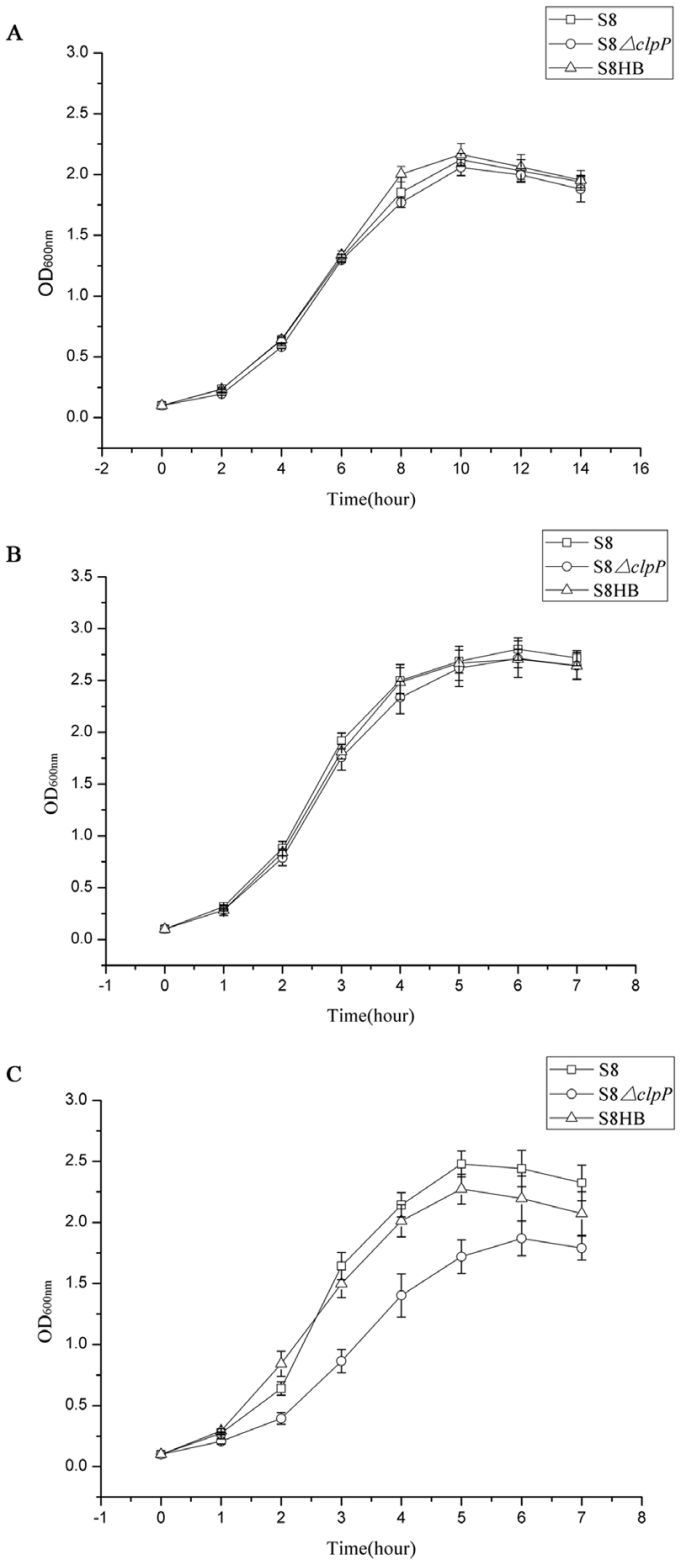
The growth curves of the *A. pleuropneumoniae* in different temperature. Overnight cultures of the S8 (□), S8*ΔclpP* (○) and S8HB (▵) strains were diluted into fresh medium and then incubated at (A) 25°C, (B) 37°C, and (C) 42°C. Growth was monitored by OD_600_ at various time points. Points indicate the mean values, and error bars indicate standard deviations.

We first examined the impact of ClpP protease on growth. As shown in Figure 1, the growth curves of the wild-type S8 strain, the S8*ΔclpP* mutant and the complemented S8HB strain were similar at 25°C and 37°C (Figure 1A and 1B), demonstrating that ClpP protease is not required for optimal growth at lower temperatures. However, the S8*ΔclpP* mutant strain exhibited impaired growth at 42°C (Figure 1C), indicating an important role for ClpP protease in the optimal growth of *A. pleuropneumoniae* at high temperatures.

### ClpP Protease is required for the stress tolerance of *A. pleuropneumoniae*

**Figure 2:**
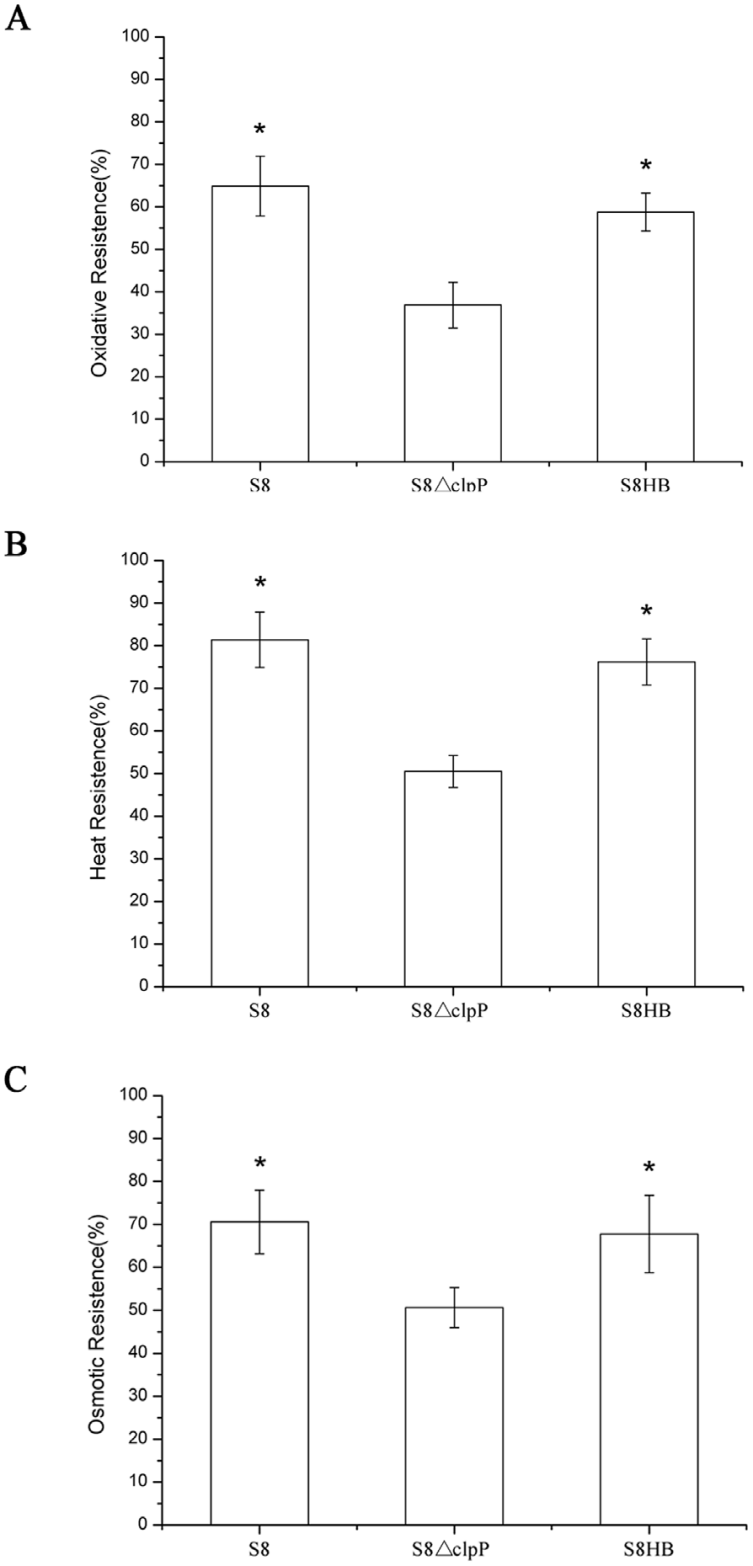
Impaired stress tolerance of the *A. pleuropneumoniae* S8*ΔclpP* mutant. Overnight cultures were inoculated into fresh medium and grown to an OD_600_ value of approximately 0.6. Cells were then treated with (A) 1 mM H_2_O_2_ for 30 min, * p<0.01, (B) 52°C heat shock for 20 min, *p<0.01, or (C) 0.3 M KCl for 1 hour, * p<0.05. * denotes *P* values (*t* test) for comparison to S8*ΔclpP*.

The wild-type S8 strain, the S8*ΔclpP* mutant and the complemented S8HB strain were exposed to various stress conditions. When the cells were treated with 1 mM hydrogen peroxide for 30 min, the S8*ΔclpP* mutant cell survival rate was 36.8%, which was much lower than that of the S8 cells (64.9%) and the S8HB cells(58.7%; Figure 2A). These results suggest that ClpP has a role in the tolerance of *A. pleuropneumoniae* to oxidative stress. Similar results were obtained in the heat shock assay. Wild-type cells incubated in a 52°C water bath for 20 min exhibited an 81.4% survival rate, and the complemented S8HB strain exhibited a 76.2% survival rate; however, only 50.5% of the S8*ΔclpP* mutant cells survived (Figure 2B). These results indicate that the deletion of *clpP* impairs the ability of *A. pleuropneumoniae* to successfully respond to heat shock. Similarly, when cells were treated with 0.3 M potassium chloride for 1 hour, the survival rate of S8*ΔclpP* mutant cells (50.6%) was lower than that of the S8 cells (70.6%) and the S8HB cells (67.8%; Figure 2C), indicating that ClpP protease is also important for the response of *A. pleuropneumoniae* to osmotic stress. Collectively, these results indicate that ClpP protease is involved in the tolerance of multiple stresses.

### ClpP Protease affects the iron acquisition ability of *A. pleuropneumoniae*

**Figure 3:**
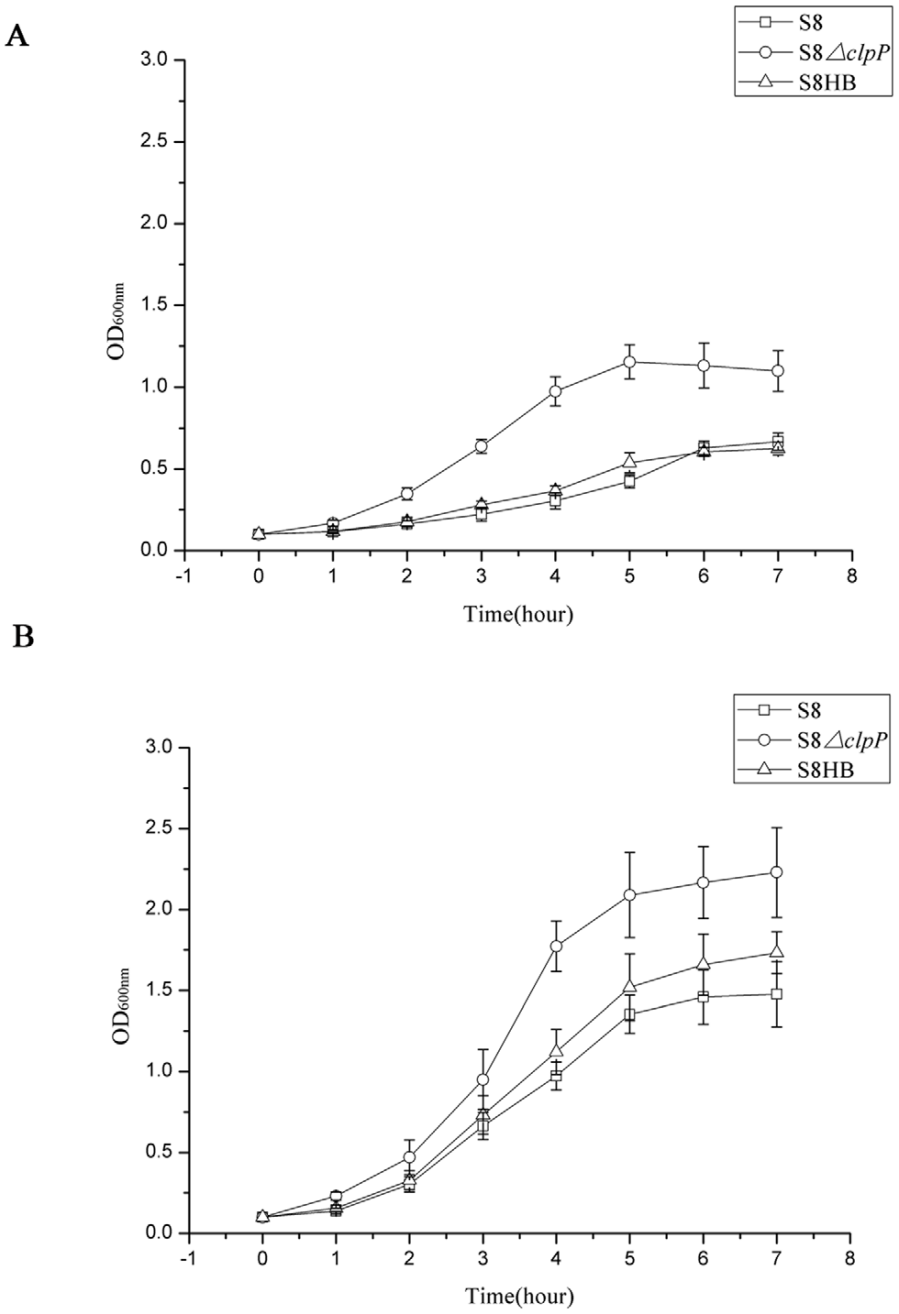
The growth curves of the *A. pleuropneumoniae* in iron-restricted and iron supplemented conditions. Overnight cultures of the S8 (□), S8*ΔclpP* (○) and S8HB (▵) strains were diluted into fresh medium and then incubated in (A) BHI containing 30 µM of the iron chelator EDDHA or (B) BHI containing 30 µM of the iron chelator EDDHA and 10 µM FeSO4. Growth was monitored by OD_600_ at various time points. Points indicate the mean values, and error bars indicate standard deviations.

The ability of the S8, S8*ΔclpP* and S8HB strains to utilize iron was analyzed using iron-restricted medium (BHI, 30 µM EDDHA) and iron supplementation medium (BHI, 30 µM EDDHA and 10 µM FeSO_4_). As shown in Figure 3A, the growth of the S8, S8*ΔclpP* and S8HB strains was significantly inhibited in low-iron, BHI medium with the addition of EDDHA. However, the S8*ΔclpP* mutant strain exhibited slightly increased growth as compared with the S8 and S8HB strains in these conditions. In the iron supplementation culture, the growth capacity of all strains was largely restored, but the growth ability of the S8*ΔclpP* mutant strain was still slightly increased relative to the S8 and S8HB strains (Figure 3B). These results suggest that the deletion of the *clpP* gene might improve the iron utilization of *A. pleuropneumoniae*.

### Loss of clpP leads to aberrant cell morphology of *A. pleuropneumoniae*

**Figure 4:**
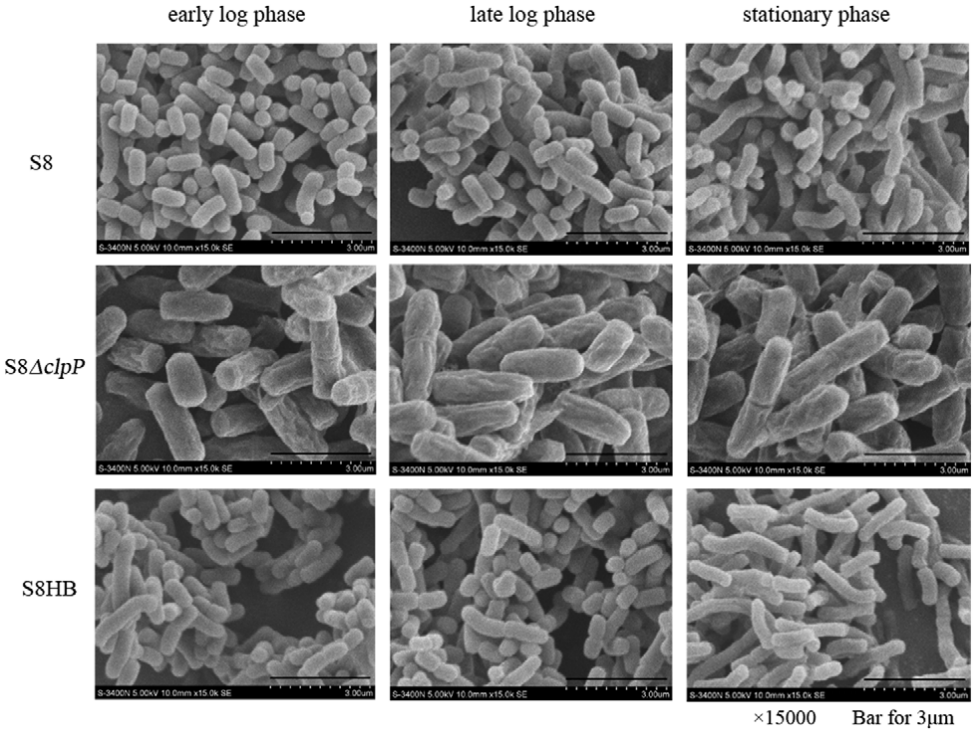
Scanning electron microscopy. SEM of S8, S8*ΔclpP* and S8HB in the early log phase, late log phase and stationary phase were carried out. Compared to the wild-type S8 strain and the complemented S8HB strain, cells of the S8*ΔclpP* mutant show increased cell volume and rougher, more irregular surfaces. Preparation of samples was performed as described in Materials and Methods.

Samples of the S8, S8*ΔclpP* and S8HB strains were processed using standard procedures and examined under a scanning electron microscope. A significant morphological variation was observed. Notably, the morphology of the S8*ΔclpP* strain showed an increase in volume (1.8-fold) compared to the wild-type S8 strain (Figure 4). Furthermore, the cells of the S8*ΔclpP* strain showed rougher, more irregular surfaces than the wild-type cells (Figure 4). However, the morphology of the complemented S8HB strain is similar to the wild-type S8 strain. These results indicate that the ClpP protease plays an important role in maintaining cell morphology related to *A. pleuropneumoniae*.

### ClpP Protease affects the biofilm formation by *A. pleuropneumoniae*

**Figure 5:**
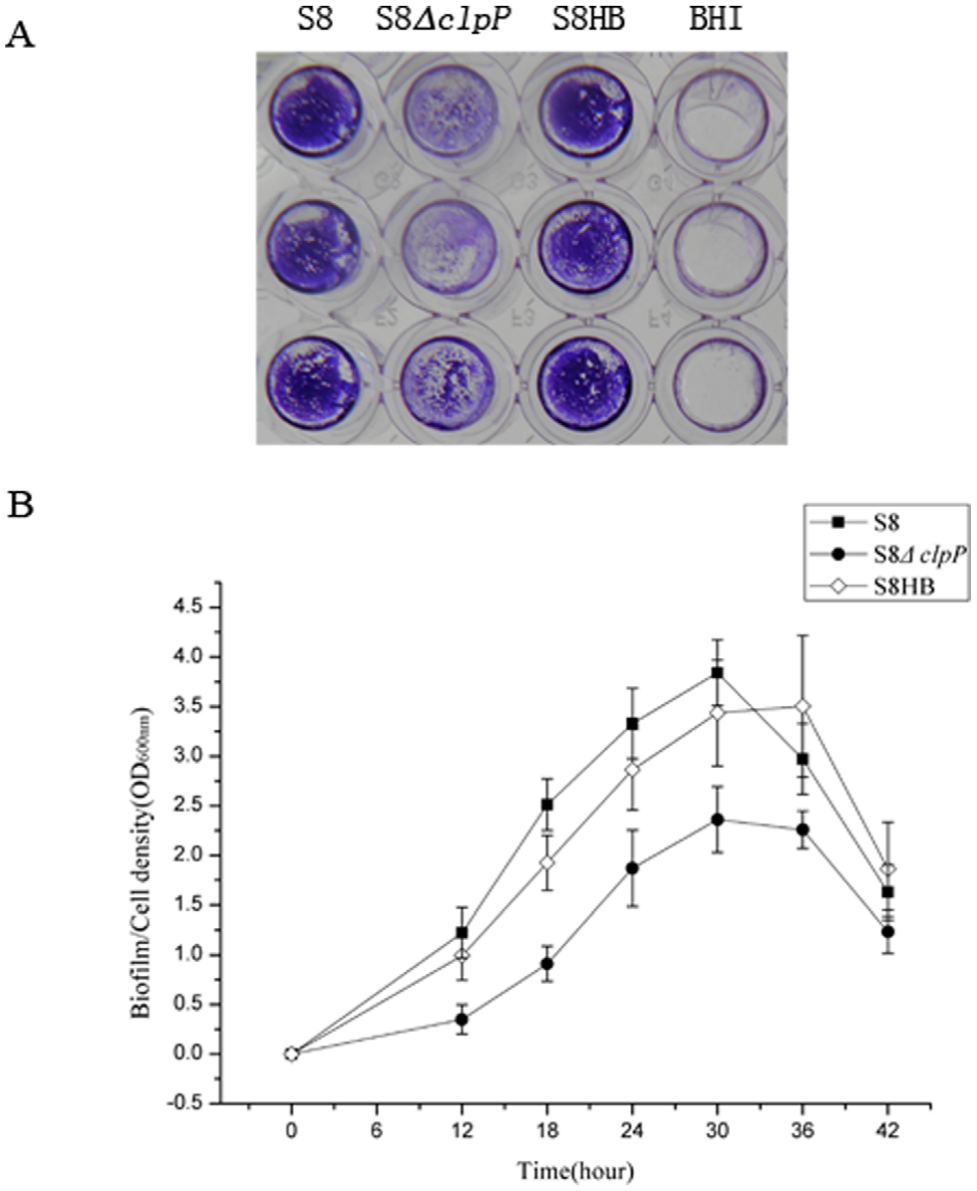
Polystyrene microtiter plate biofilm assay. (A) Biofilm formation of the S8, S8*ΔclpP* and S8HB strains in the wells of 96-well polystyrene microtiter plates. The plates were stained with crystal violet. (B)The quantitative determination of biofilm formation. The S8 (▪), S8*ΔclpP* (•) and S8HB (⋄) strains were grown in BHI supplemented with NAD. The optical density of the bacterial biofilm formation was monitored by OD_600_ after 12, 18, 24, 30, 36 and 42 h of incubation. Points indicate the mean values, and error bars indicate standard deviations.

**Figure 6:**
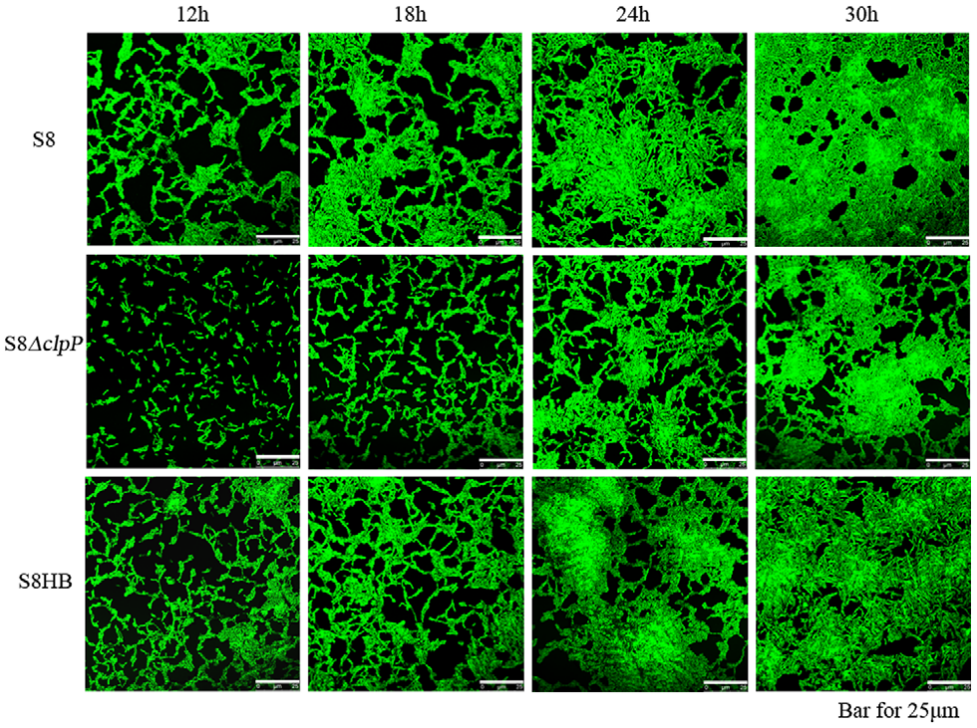
Microscopy analysis of the biofilm formation. Biofilm development was monitored by confocal scanning laser microscopy (CSLM) after 12, 18, 24, and 30 h. The cells were stained with LIVE/DEAD^@^ BacLight^Tm^ Bacterial Viability Kit solution. The S8*ΔclpP* strain shows a reduction in biofilm formation compared to the wild-type S8 strain and the complemented S8HB strain.

The biofilm formation phenotype of the S8, S8*ΔclpP* and S8HB strains was examined in polystyrene microtiter plates using crystal violet staining (Figure 5A) and was quantitatively analyzed using a microplate reader (Figure 5B). The S8*ΔclpP* mutant exhibited weak biofilm formation, while the biofilm formation phenotypes of the S8 and S8HB strains were stronger than the S8*ΔclpP* phenotype. The biofilm formation process was also observed under a confocal scanning laser microscope (Figure 6). Overall, the biofilm formation was significantly decreased during the middle to late exponential phases in the S8(clpP mutant strain compared to the S8 and S8HB strains under each culture condition (Figure 5 and 6). The clpP mutation attenuates biofilm formation in this strain, indicating that ClpP protease is required for biofilm formation in A. pleuropneumoniae.

### Differential expression analysis

To identify the A. pleuropneumoniae genes affected by the deletion of the *clpP* gene, the S8*ΔclpP* and S8 strains were transcriptionally profiled using RNA sequencing. A total of 13,694,332 and 12,883,314 reads were obtained for each library (“S8*ΔclpP*” and “S8”, respectively). Of these reads, 13,340,847 (S8*ΔclpP*) and 12,589,286 (S8) reads mapped to the genome, and 13,316,197 (S8*ΔclpP*) and 12,562,203 (S8) mapped uniquely. One thousand two hundred thirteen of the unique reads in the S8 sample aligned to the *clpP* gene, while none of the reads from the S8*ΔclpP* sample aligned to this gene. This finding proved that the *clpP* gene was not expressed in the S8*ΔclpP* mutant. The differential expression analysis of the S8*ΔclpP* and S8 strains revealed that 16 *A. pleuropneumoniae* genes exhibited an FDR≤0.001 and an absolute value of log2Ratio absolute value ≥1, proving that these genes were affected by the deletion of the *clpP* gene (Table 2). Of these 16 genes, 4 were upregulated, including 2 genes involved in the iron acquisition system, and 12 were downregulated.

**Table 2:** The regulated genes in *A. pleuropneumoniae* by the deletion of the *clpP* gene.

Gene	Log2 Ratio	Annotation
**Up-regulated genes**		
APP7_1881	1.46581	putative periplasmic iron/siderophore binding protein
APP7_1156	1.198833	hypothetical protein
APP7_1879	1.051793	Fe(III) dicitrate ABC transporter, ATP-binding protein
APP7_0104	1.037253	hypothetical protein
**Down-regulated genes**		
APP7_0616	−2.30679	pyridoxal biosynthesis lyase PdxS
APP7_2064	−2.12199	tRNA 2-thiouridine synthesizing protein A
APP7_1497	−1.78015	hypothetical protein
APP7_0617	−1.5762	glutamine amidotransferase subunit PdxT
APP7_0418	−1.44626	RNA polymerase sigma-70 factor
APP7_0419	−1.33961	putative sigma-E factor negative regulatory protein
APP7_1517	−1.26523	hypothetical protein
APP7_1695	−1.25314	hypothetical protein
APP7_1286	−1.1338	maltose/maltodextrin import ATP-binding protein MalK
APP7_1284	−1.0894	maltose operon periplasmic protein
APP7_0747	−1.05714	putative methylation subunit, type III restriction-modification system
APP7_1152	−1.04195	hexosaminidase

## Discussion

The ClpP protease is a conserved protein that is present in many bacteria [32]. In this study, we initially determined the nucleotide level similarities in the *clpP* gene sequences with a BLASTN analysis. Currently, only the complete genomes of *A. pleuropneumoniae* serotypes 3, 5 and 7 have been sequenced. We found that the *clpP* gene was present in all of these genomes and that the identity of these nucleotide sequences was more than 94%. We also used PCR to confirm that the *clpP* gene is present in *A. pleuropneumoniae* serotypes 1–12 reference strains and 11 *A. pleuropneumoniae* isolates available in our laboratory (data not shown). These results indicated that the *clpP* gene is conserved in several *A. pleuropneumoniae* serotypes. In the present study, the *clpP* mutant was constructed and challenged with a variety of stressors and growth conditions to examine the physiological role of the ClpP protease in the stress tolerance, cell morphology, and biofilm formation related to *A. pleuropneumoniae*. To confirm that the characteristics of the S8*ΔclpP* mutant were not due to polar effects and were truly caused by the *clpP* gene deletion, we also constructed a complemented strain S8HB. In addition, the RNA sequencing results indicated that the expression levels of the 10 genes upstream of the *clpP* gene and the 10 genes downstream of the *clpP* gene did not change. All of these results proved that the characteristics of the S8*ΔclpP* mutant were truly caused by the deletion of the *clpP* gene and not by polar effects.

In this study, we observed that the S8*ΔclpP* mutant exhibited a reduced growth rate at high temperatures (Figure 1C) and an impaired resistance to heat shock (Figure 2B) compared to the wild-type S8 strain and the complemented S8HB strain. Several previous studies have demonstrated growth deficits resulting from ClpP deficiency over a broad range of temperatures [7], [33], [34]. However, there was no significant difference between the growth curves of the S8*ΔclpP* mutant, the wild-type S8 strain and the complemented S8HB strain when these strains were cultured at a lower temperature (Figure 1A). The observations in our study suggest a more restricted role for ClpP in *A. pleuropneumoniae*, independent of cold stress.

Iron is an essential factor for the growth of *A. pleuropneumoniae*, and low iron availability in the host represents a major stress for the pathogen. *A. pleuropneumoniae* has evolved a highly sophisticated system for iron acquisition that includes transferrin receptor complexes TbpA/TbpB, TonB-ExbB-ExbD, Afu, ABC transporter and so on [26]. In the current study, the S8*ΔclpP* mutant was shown to exhibit a faster growth compared to the wild-type S8 strain and the complemented S8HB strain. In contrast, this result was not reported in studies on the role of ClpP in other bacteria. Based on this result, we hypothesize that the ClpP protease might regulate the expression of some of the genes involved in iron uptake, thus regulating virulence. Therefore, we also transcriptionally profiled the effects of the deletion of the *clpP* gene in *A. pleuropneumoniae*. The results of this analysis showed that 2 genes encoding a putative periplasmic iron/siderophore binding protein and a Fe(III) dicitrate ABC transporter were upregulated. This finding indicated that the inactivation of the ClpP protease affects the expression of these two iron acquisition proteins.

Bacterial biofilm formation is a complex, multifactorial process requiring genes involved in adherence, metabolism, quorum sensing, and the stress response [35]. It has been shown that several genes involved in these factors mentioned above affect the biofilm formation by *A. pleuropneumoniae*, including the genes coding for polysaccharide PGA [36]; ArcA, which is a regulator involved in anaerobic metabolism [37]; and LuxS, which is a regulator involved in quorum sensing [38]. This study is the first to show the role of stress protein in *A. pleuropneumoniae* biofilm formation. We found that the *clpP* mutation slowed down biofilm formation. In addition, the similar growth rates of the S8 and S8*ΔclpP* strains at 37°C (Figure 1B) suggest that it is unlikely that the reduced biofilm formation observed in the *clpP* mutant is due to the inherently different growth rates between strains. The results of the RNA sequencing analysis indicated that a gene encoding hexosaminidase was downregulated in the S8*ΔclpP* mutant. It has been shown that hexosaminidase removes the terminal residues from glycoproteins and exposes β-linked glucosamine, thus mediating biofilm formation in *A.pleuropneumoniae* [36], [39]. Our findings indicate that the ClpP protease regulates the expression of the gene encoding hexosaminidase, thus contributing to the attenuation of biofilm formation. In addition, we also found that the expression of some genes encoding hypothetical proteins was upregulated or downregulated. These changes might also affect biofilm formation in *A. pleuropneumoniae*. However, these findings should also be confirmed in future studies.

## Supporting Information

Figure S1 **Schematic representation of the** ***A. pleuropneumoniae clpP*** **locus.** The figure shows the binding locations for the oligonucleotide primers used to amplify the two flanking regions (1249 bp and 1200 bp, respectively) used in the construction of the pEM*ΔclpP* plasmid and the diagnostic PCR analysis of the *clpP*-deleted mutant (367 bp) and wild type *A. pleuropneumoniae* strains (858 bp). The S8*ΔclpP* mutant contains a 491 bp in-frame deletion (shadowed domain) in the *clpP* gene.
(TIF)

Figure S2 **PCR identification of the S8** ***ΔclpP*** **mutant.** PCR identification of the S8*ΔclpP* mutant using the paired primers clpPJDF/clpPJDR. For lanes 8, the identified S8*ΔclpP* mutant (367 bp); for lane M, DL2000 DNA marker was used (from top to bottom: 2000, 1000, 750, 500, 250, and 100 bp); for other lanes, the wild-type S8 strain.
(TIF)
