# Correction: The ClpP Protease Is Required for the Stress Tolerance and Biofilm Formation in *Actinobacillus pleuropneumoniae*

**DOI:** 10.1371/annotation/fbe62755-866e-4a4e-baf8-4bb40019a8fb

**Published:** 2013-08-07

**Authors:** Fang Xie, Yanhe Zhang, Gang Li, Long Zhou, Siguo Liu, Chunlai Wang

Two grants and their numbers were incorrectly omitted from the Funding statement. The grants and their numbers are: the Special Fund for Agro-scientific Research in the Public Interest (No. 201303034) and the Special Fund of Harbin Science and Technology for Young Outstanding Innovative Talents (No. 2012RFLYN009). 

