# Supplementary figures and images for: The ClpP Protease Is Required for the Stress Tolerance and Biofilm Formation in Actinobacillus pleuropneumoniae
Source: PLoS One. 2013 Jan 11;8(1):e53600. doi: 10.1371/journal.pone.0053600 (PMC3543445; doi:10.1371/journal.pone.0053600)

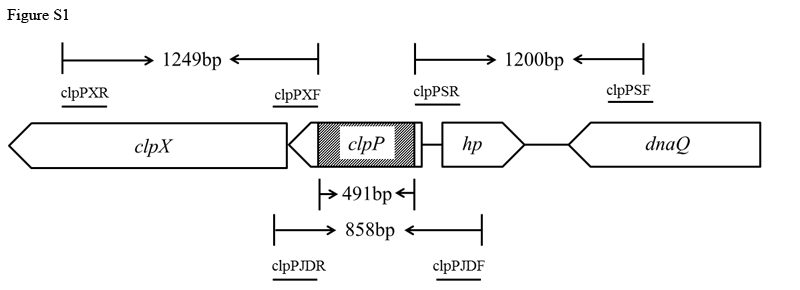

Supplement: Figure S1 — Schematic representation of the A. pleuropneumoniae clpP locus. The figure shows the binding locations for the oligonucleotide primers used to amplify the two flanking regions (1249 bp and 1200 bp, respectively) used in the construction of the pEMΔclpP plasmid and the diagnostic PCR analysis of the clpP-deleted mutant (367 bp) and wild type A. pleuropneumoniae strains (858 bp). The S8ΔclpP mutant contains a 491 bp in-frame deletion (shadowed domain) in the clpP gene. (TIF) [file pone.0053600.s001.tif]

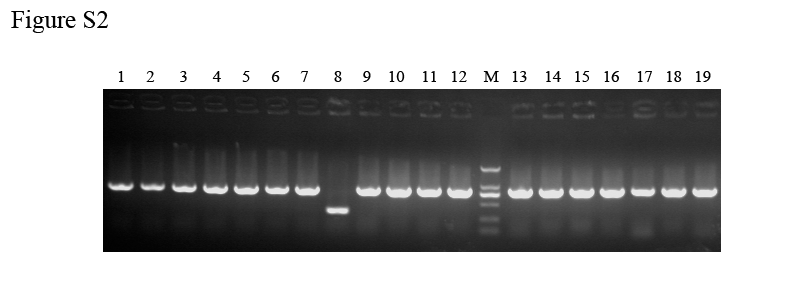

Supplement: Figure S2 — PCR identification of the S8 ΔclpP mutant. PCR identification of the S8ΔclpP mutant using the paired primers clpPJDF/clpPJDR. For lanes 8, the identified S8ΔclpP mutant (367 bp); for lane M, DL2000 DNA marker was used (from top to bottom: 2000, 1000, 750, 500, 250, and 100 bp); for other lanes, the wild-type S8 strain. (TIF) [file pone.0053600.s002.tif]
